# Fear of falling and cognitive impairment in elderly with different social support levels: findings from a community survey in Central Vietnam

**DOI:** 10.1186/s12877-020-01533-8

**Published:** 2020-04-16

**Authors:** Thi Hue Man Vo, Keiko Nakamura, Kaoruko Seino, Hoang Thuy Linh Nguyen, Thang Van Vo

**Affiliations:** 1 Department of Global Health Entrepreneurship, Division of Public Health, Graduate School of Medical and Dental Sciences, Tokyo Medical and Dental University, Yushima 1-5-45, Bunkyo-ku, Tokyo, 113-8519 Japan; 2 Faculty of Public Health, College of Medicine and Pharmacy, Hue University, Hue, Vietnam; 3 The Institute for Community Health Research, College of Medicine and Pharmacy, Hue University, Hue, Vietnam

**Keywords:** Fear of falling, Cognitive impairment, Social support, Elderly

## Abstract

**Background:**

Fear of falling (FoF) in the elderly is one of the major public health concerns in this era of aging of the population. As there is limited evidence on how cognitive function may differ by social support level in relation to FoF among the elderly, this cross-sectional study aims to investigate the prevalence of FoF and the associations between cognitive impairment and FoF by the social support level, after adjustments for potential confounders.

**Methods:**

Data from the “Health needs assessment of elderly in Thua Thien Hue Province, Vietnam in 2018” survey of 725 elderly aged 60 years or older were used for analysis. FoF was assessed using the Fall Efficacy Scale-International. High FoF was defined as a score above 28. The Multidimensional Scale of Perceived Social Support was used to measure the perception of support. Logistic regression analysis was performed to investigate the association between cognitive function and FoF by social support levels (*p* < 0.05).

**Results:**

The prevalence of high FoF among the elderly was 40.8%. Female gender, advanced age, a marital status of single or formerly married, living alone, history of injury, history of falls, chronic diseases (arthritis and/or hypertension), limitations of the IADL and BADL, visual difficulty and walking difficulty, low social support, and cognitive impairment were all significantly associated with a high FoF. After adjustments for the age, gender, marital status, history of falls and health-related factors, cognitive impairment remained significantly associated with a high FoF among the elderly with a low to moderate social support level (OR = 2.97, 95% CI 1.49–5.89), but not in those with a high social support level.

**Conclusions:**

A high FoF was associated with impairment of cognitive function among the elderly who perceived themselves as having low or moderate support levels, even after adjustments for socio demographic and physical functional factors. However, this association was not observed among the elderly who perceived themselves as having high social support levels. Fall prevention programs for the elderly with various levels of social support should be carefully devised, keeping in mind the cognitive function levels of the target recipients.

## Background

Globally, falls account for 40% of all deaths from injuries, and affect one-third of the elderly population each year [1]. Fatal falls rates are highest among adults over the age of 60 years [1].

Considering the implications of falls in older persons, fear related to falls should also not be neglected. Fear of falling (FoF), defined as a lasting concern about falling that leads to avoidance of daily activities [2], is also a highly prevalent problem [3]. FoF increases with age and is associated with adverse consequences among the elderly, including a poor quality of life [4], mental and functional decline [4–8], a lower level of satisfaction with life [9], and restricted social interactions [7, 10]. As falls and FoF are closely linked with each other by shared predictors, each is a risk factor for the other, and FoF was reported to be an independent predictor of falls [11], recent research has been focused on investigating the determinants of FoF, in an effort to prevent both FoF and falls in the community-dwelling elderly.

A number of studies have already been published on the FoF and its risk factors in the elderly. Although cognitive impairment has frequently been mentioned a factor that is significantly associated with the FoF, reports on the relationship between cognitive impairment and FoF are conflicting. In some previous studies, impaired cognition was identified as a predictor of FoF [4, 5, 12], while in others, no association between cognitive impairment and FoF was observed [13]. In addition, the proportion of Vietnamese elderly who had cognitive symptoms of dementia was recently reported to be high at 46.4% [14]. As there have been limited large-scale population studies investigating FoF as well as the relationship between FoF and cognitive impairment in Vietnam, it is crucial to obtain a clearer understanding about this association in a large community-based sample.

Social support is well-known to play a vital role in maintaining well-being in old age [15], however, few studies have investigated the difference in the relationship between cognitive function and FoF according to the level of social support available to the elderly. Since higher cognitive function is known to be associated with a higher level of social support [16], and good social support is known to be associated with a lower odds of the FoF [17–19], the level of social support available may have influence on the association between cognitive impairment and FoF.

In developing countries where the population is rapidly ageing, fall-related issues have yet to receive adequate attention. Vietnam is among the countries with fastest rates of ageing in Asia [20] and it is predicted that the proportion of persons aged 60 years old or older will reach 20% of the total population by the year 2038 [21]. A community survey in 2015 showed that the prevalence of high FoF (FES-I scores ranged from 28 to 64) among Vietnamese elderly was high, at 64% [6]. Although the risk factors of FoF have been widely explored, there are many aspects of FoF that still remain to be investigated further, and how factors influencing the FoF might have different levels of influence depending on the social support available to the elderly remains unknown in the context of Vietnam.

Thus, this study was performed to investigate the prevalence of FoF and the differential associations between cognitive impairment and FoF depending on the social support level in a community of elderly in Vietnam.

## Methods

### Study area

The study was performed in Thua Thien Hue Province, Central Vietnam. The province comprises 9 districts and 152 communes with a total population of 1.154 million people as of a census conducted in 2017 [22].

### Sample size and sampling method

In Vietnam, people aged 60 years and over are considered elderly [23]. Sample size needed was calculated as 726 by the standard formula for cross-sectional studies with a 95% confidence level, a 5% margin of error, a design coefficient of 2 and *p* = 0.38 based on an estimation of the proportion of the elderly who had at least one difficulty in daily life activities from the previous study [24]. According to this calculation, 730 participants from Hue city and Phu Vang district were recruited by a two-stage random cluster sampling method. In the first stage, two quarters among the 27 quarters of Hue city (urban area) and three communes out of 20 communes in Phu Vang district (rural area) were randomly selected. In the second, in each of the selected quarters and communes, 146 households with people aged 60 years old or over were randomly selected based on a list of households held in the commune health centers.

### Data collection

A cross-sectional study was conducted in the elderly individuals living in their own households in Thua Thien Hue Province between June and July 2018. Face to face interview using a structured questionnaire was performed to collect data from the selected households. We excluded participants who refused to take part in the survey or were unable to speak. Data of a total of 725 participants were finally included in the analysis, representing a response rate of 99% (725/730).

### Survey instruments

The data collection instrument was adapted from a reference questionnaire developed by a research team from the Tokyo Medical and Dental University, and containing questions pertaining to the socio–demographic characteristics, physical functional status, FoF, perceived social support and cognitive function of the participants. The instrument was translated from English into Vietnamese and then translated back to English by two Vietnamese researchers to check for the accuracy of the translation. The Vietnamese questionnaire was pilot-tested with 20 elderly living in community in Thua Thien Hue province. A few modifications in wording in questions were made to improve comprehensibility and to ensure the cultural relevance in Vietnam.

### Ethical consideration

Ethical approval was obtained from the Institutional Ethics Committee of Hue University of Medicine and Pharmacy (Ethics Approval No. H2018/148 dated 12 May 2018). Written informed consent was obtained from each of the participants or from their guardian prior to the interviews. Study ID numbers were assigned to all the participants to protect their confidentiality.

### Study variables

#### Fear of falling

FoF was assessed using the Falls Efficacy Scale-International (FES-I), which contains 16 items. The overall scores can range from 16 to 64, with higher scores indicating a higher FoF. Scores of 16–19 represent low levels of concern; scores of 20–27 represent moderate levels of concern, and scores of 28–64 represent high levels of concern. Good validity and reliability of FES-I have been demonstrated in a previous study [25].

#### Perceived social support

Perceived social support was measured by The Multidimensional Scale of Perceived Social Support (MSPSS), to assess the participants’ perception of support from three sources: family, friends and significant other. The scale is comprised of 12 items, with 4 items for each category scored on a seven-point Likert scale ranging from ‘very strongly disagree’ to ‘very strongly agree’. The total scores range from 12 to 84. Mean total scores in the range of 1.0 to 2.9 represent low levels of support, scores of 3.0 to 5.0 represent moderate levels of support, and scores of 5.1 to 7.0 represent high levels of support [26]. The good validity, high internal consistency and reliability of MSPSS has been shown in a previous study [26].

#### Cognitive function

The Mini-Mental State Examination (MMSE) was used to evaluate the cognitive function. Cognitive impairment was defined as a score lower than the cutoff point of 23, which is the most commonly used cutoff score to detect cognitive impairment [27].

#### Functional limitation

Performance in the activities of daily living was assessed by the Barthel ADL index and Lawton Instrumental Activities of Daily Living Scale (IADL), as follows:

- The IADL scale measured eight domains of function: use of telephone, shopping, food preparation, housekeeping, laundry, mode of transportation, medication, and managing finance with the possible summary score ranging from 0 (low function) to 8 (high function).
- The Barthel ADL scale pertained to 10 items: feeding, bathing, grooming, dressing, bowels, bladder, toilet use, transfers, mobility, and use of stairs. Response categories were scored based on each item and total possible score ranges from 0 to 20.

Higher total scores in both scales indicate a greater level of independence. Total independence was categorized as ‘no limitation’, and partial dependence and complete dependence were categorized as ‘limitation’ [28, 29].

#### Socio-demographic characteristics and physical functional status

The questionnaire consisted of a variety of socio-demographic characteristics, including the age, gender, residential area, education level, marital status and living arrangement. Physical functional status variables included experience of injury, chronic diseases (arthritis, hypertension and others), functional limitation status, visual ability and walking ability.

### Data analysis

Data were summarized using descriptive analysis and differences in the levels of FoF by the participants’ socio-demographic, physical functional status and cognitive function characteristics. Physical functional status included experience of injury, history of falls, arthritis, hypertension, limitation of IADL and BADL, visual ability and walking ability. A Chi-square test was used to assess the association between the three groups of FoF and above mentioned parameters. As scores were not normally distributed, the overall FES-I score was categorized. For the analysis with logistic regression model, the dependent variable “Fear of falling” was categorized as “high FoF” and “not high FoF” (combining the “Low FoF” and “Moderate FoF” groups). The associations between the independent variables and the dependent variable “Fear of falling” were investigated by bivariate analysis. Multivariate logistic regression analysis was used to examine the association between cognitive impairment and FoF, after adjustments for socio-demographic factors and the physical functional status, by adjusted odd ratios with 95% confidence intervals. Data analysis was performed using SPSS version 22. *P*-values of less than 0.05 were considered as indicative of statistical significance.

## Results

The characteristics of participants by three levels of FoF were shown in Table 1. The total sample size was 725 elderly persons aged 60 or over, of whom 423 were females. The prevalence of history of fall, hypertension, arthritis, diabetes and stroke were 7.4, 43.9, 33.5, 10.5 and 4%, respectively. The prevalence of elderly with a high FoF was 40.8%. The overall prevalence of a high FoF was greater among the cognitive-impaired elderly than among those with normal cognition (76.4 and 33.6%, *p* < 0.001).

**Table 1:** Baseline characteristics of the participants according to fear of falling status

Characteristics of participants		All (*n* = 725)	Low FoF (*n* = 272)	Moderate FoF (*n* = 157)	High FoF (*n* = 296)	*P* value^1^
			%	%	%	
**Socio-demographic characteristic**						
Gender	Female	423	28.1	20.8	51.1	< 0.001
	Male	302	50.7	22.8	26.5	
Age (yr)	60–69	340	51.2	21.5	27.4	< 0.001
	70–79	209	30.6	25.8	43.5	
	≥ 80	176	39.3	17.0	63.6	
Residential area	Hue City	443	38.8	23.0	38.1	0.173
	Phu Vang District	282	35.5	19.5	45.0	
Marital status	Married	524	42.4	22.5	35.1	< 0.001
	Single or formerly	201	24.9	19.4	55.7	
	married					
Education level	Low education	195	22.6	16.4	61.0	< 0.001
	Primary to high school	468	42.3	23.3	34.4	
	College and higher	55	52.7	25.5	21.8	
Living arrangement	Alone	65	24.6	15.4	60.0	0.004
	Living with others	660	38.8	22.3	38.9	
**Physical functional status**						
Experience of injury	No	665	39.7	22.6	37.7	< 0.001
	Yes	60	13.3	11.7	75.0	
History of fall	Yes	54	14.8	7.4	77.8	< 0.001
	No	671	39.3	22.8	37.9	
Hypertension	Yes	318	32.7	21.1	46.2	0.022
	No	407	41.3	22.1	36.6	
Arthritis	Yes	243	30.9	18.9	50.2	0.001
	No	482	40.9	23.0	36.1	
Limitation of the IADL	No	484	50.6	24.8	24.6	< 0.001
	Yes	241	11.2	15.4	73.4	
Limitation of the BADL	No	598	43.5	24.7	31.8	< 0.001
	Yes	127	9.4	7.1	83.5	
Visual ability	No difficulty	370	50.3	21.9	27.8	< 0.001
	Have difficulty	355	24.2	21.4	54.4	
Walking ability	No difficulty	503	48.3	26.6	25.0	< 0.001
	Have difficulty	222	13.1	10.4	76.6	
Social support	Low	71	25.4	16.9	57.7	0.002
	Moderate	352	35.8	26.1	38.1	
	High	302	42.4	17.5	40.1	
Cognitive function	Normal	602	43.2	23.3	33.6	< 0.001
	Impairment	123	9.8	13.8	76.4	

^1^P-value of Chi-square test for the three group of FoF and characteristics of participants

Table 2 showed the results of bivariate analysis, which revealed the existence of an association between the participants’ characteristics and the degree of FoF. Factors that were significantly associated with a high FOF among all subjects included: female gender, age 80 years or over, marital status of being single or formerly married, living alone, and a low social support level (*p* < 0.05). A positive history of fall was associated with a higher likelihood of high FoF among the elderly (OR: 5.75, 95% CI: 2.97–11.12). Physical functional status variables, including experience of injury in the last 12 months, presence of arthritis and hypertension; limitations of IADL and BADL; visual and walking ability showed significant association with a high FoF (*p* < 0.01).

**Table 2:** Associations between participants’ characteristics and FoF, unadjusted logistic analysis (*n* = 725)

		Fear of falling	
		OR	95% CI
**Socio-demographic characteristic**			
Gender	Male	1	
	Female	2.90***	(2.11–3.89)
Age (yr)	60–69	1	
	70–79	2.05***	(1.43–2.94)
	≥ 80	4.65***	(3.15–6.86)
Residential area	Hue city	1	
	Phu Vang District	1.33	(0.98–1.80)
Marital status	Married	1	
	Single or formerly married	2.33***	(1.67–3.24)
Education level	College and higher	1	
	Low education	5.61	(2.78–11.32)
	Primary to high school	1.88	(0.96–3.66)
Living arrangement	Living with others	1	
	Alone	2.35**	(1.40–3.96)
**Physical functional status**			
Experience of injury	No	1	
	Yes	4.95***	(2.70–9.06)
History of fall	No	1	
	Yes	5.75***	(2.97–11.12)
Arthritis	No	1	
	Yes	1.79***	(1.31–2.44)
Hypertension	No	1	
	Yes	1.49**	(1.11–2.01)
Limitation of the IADL	No	1	
	Yes	8.48***	(5.96–12.07)
Limitation of the BADL	No	1	
	Yes	10.84***	(6.58–17.85)
Visual ability	No difficulty	1	
	Have difficulty	3.09***	(2.27–4.21)
Walking ability	No difficulty	1	
	Have difficulty	9.78***	(6.75–14.17)
Social support	High	1	
	Low	2.04**	(1.21–3.45)
	Moderate	0.92	(0.67–1.26)
Cognitive function	Normal	1	
	Impairment	6.42***	(4.10–10.06)

*OR* Odds Ratio; 95% CI = 95% Confidence interval

*p < 0.05; **p < 0.01; ***p < 0.001

As shown in Table 3, after adjustments for socio - demographic factors and the physical functional status, elderly with impaired cognition had an approximately 3 times higher likelihood of having a high FoF as compared to those without cognitive impairment among those with low and moderate perceived levels of social support (AOR: 2.97, 95% CI: 1.49–5.89). In contrast, in the group with a high perceived social support level, cognitive impairment was not significantly associated with a high FoF. Female participants with low and moderate perceived levels of social support were 1.9 times more likely to have a high FoF as compared to males of the same group (AOR: 1.99, 95% CI: 1.10–3.59). History of fall, limitation of the IADL, limitation of the BADL and difficulty in walking were statistically significant associated with a high FoF in the entire sample.

**Table 3:** Associations between participants’ characteristics and FoF by social support level, adjusted logistic analysis (*n* = 725)

		Fear of falling	
		Model 1 Low - moderate social support level (*n* = 423)	Model 2 High social support level (*n* = 302)
		AOR (95% CI)^1^	AOR (95% CI)
Gender	Male	1	1
	Female	1.99 (1.10–3.59)*	1.91 (0.93–3.92)
Age (yr)	60–69	1	1
	70–79	1.13 (0.63–2.02)	1.61 (0.77–3.33)
	≥ 80	1.55 (0.77–3.10)	1.79 (0.73–4.40)
Marital status	Married	1	1
	Single or formerly married	1.23 (0.72–2.12)	0.62 (0.25–1.53)
History of fall	No	1	1
	Yes	3.36 (1.30–8.68)*	4.61 (1.09–19.57)*
Arthritis	No	1	1
	Yes	1.36 (0.80–2.33)	0.82 (0.41–1.62)
Hypertension	No	1	1
	Yes	0.62 (0.37–1.01)	1.06 (0.56–1.99)
Limitation of the IADL	No	1	1
	Yes	2.40 (1.31–4.39)**	3.41 (1.51–7.71)**
Limitation of the BADL	No	1	1
	Yes	2.35 (1.11–4.98)*	4.68 (1.56–14.03)**
Visual ability	No difficulty	1	1
	Difficulty	1.40 (0.84–2.33)	1.73 (0.94–3.21)
Walking ability	No difficulty	1	1
	Difficulty	2.58 (1.37–4.86)**	4.62 (2.17–9.82)***
Cognitive function	Normal	1	1
	Impairment^2^	2.97 (1.49–5.89)**	1.04 (0.38–2.82)
Pseudo R^2^		0.31	0.36

^1^*AOR* Adjusted odd ratio; 95% CI = 95% Confidence interval

Adjusted for age, gender, marital status, history of fall, arthritis, hypertension, limitation of the IADL, limitation of the BADL, visual ability, walking ability and cognitive function

^2^ Cognitive impairment was defined by a score lower than the cut-off score of 23

*p < 0.05; **p < 0.01; ***p < 0.001

## Discussion

The results of the current study showed that 40.8% of the participants had a high FoF. The factors associated with a high FoF varied with the perceived level of social support. Among the participants with perceived low and moderate levels of social support, cognitive impairment was associated with a high FoF, whereas in the participants with a high perceived level of social support, this relationship of the FoF with cognitive function was not observed.

### Prevalence of high FoF

This study conducted in a population-representative sample of 725 Vietnamese elderly living in rural and urban areas revealed a high prevalence of FoF. Overall, 40.8% of the elderly were identified as having a high FoF. This considerably high prevalence as compared to previous reports [9, 30, 31] might need to be interpreted from the perspective of the study location [18, 32], age of the participants (mean age: 72 ± 8.8) and standardized measurement of the FoF [3]. While safe environment could have effects on the FoF [18, 32], another possible reason is Thua Thien Hue’s long rainy season, which normally lasts for 4 months, from September to December, with a large amount of rainfall [33], which leaves surfaces slippery and unsafe for the elderly to perform their daily activities. This finding suggests the high level of concern for falls among Vietnamese elderly living in the community. Therefore, attention should be paid to this population in terms of falls and fall-related issues.

### Cognitive impairment and FoF among different perceived social support levels

The univariate analyses in the present study indicated that elderly persons with impaired cognition had a higher odd of experiencing a high FoF. With advancing age, attention and memory are the two aspects of cognition that are the most severely affected [34]. Impairment of cognitive function in the elderly, characterized by a poorer performance in memory, executive functions, attention and information processing, has been reported as a predictor of the onset of FoF [5] and to also be associated with higher levels of anxiety [35], which, in turn, was shown to be associated with a FoF [36]. Furthermore, in the logistic regression model with adjustments for confounding factors performed to determine the association between the participant’s characteristics and the likelihood of a high FoF (Additional file 1), elderly with cognitive impairment were approximately 2 times more likely to have a high FoF than those with normal cognition. This finding confirmed the effect of cognitive impairment on the high FoF among the study participants.

An even stronger association between cognition and FoF was observed among the elderly with low and moderate perceived levels of social support in the fully adjusted model: elderly with cognitive impairment in this group had a nearly 3 times higher likelihood of having high a FoF. This result can be explained by the observed characteristics of the study participants with two different perceived levels of social support, as well as by the two-way relationship between the perceived social support level and the fear of falling. A lower perceived social support level among the Vietnamese elderly might be one of the consequences of social modernization, which is currently spreading quickly in Vietnam; the younger generation tends to migrate to bigger cities and leave older persons on their own [37]. Moreover, Vietnamese elderly are exposed to less social interactions due to the limited social activities held by the local authority as compared to other developed countries [6]. Elderly with lower levels of social support are more likely to be physically inactive [38], depressed [36, 39, 40], which are reported to be closely related to FoF [18, 41]. In addition, we found that the mean MMSE score of the participants with low-moderate perceived social support levels was 25.5, which was significantly lower than that of those with a high perceived level of social support (26.6) (Additional file 2). This was also in line with a prior finding in an Asia setting of a positive relationship between better cognitive function and a higher social support level [16]. Accordingly, an ageing-friendly environment, such as the availability of wide sidewalks, a proper rainwater drainage system, and establishment of more social activities for the elderly in the community may represent some solutions to prevent FoF, especially in the elderly with limited social support and impaired-cognition.

However, this association between cognitive decline and the FoF was no longer seen in participants with perceived high social support levels. Considering the close-knit community culture in Thua Thien Hue province, older adults with a high perceived level of social support are more likely to be surrounded by their families, neighbors and/or friends, which is beneficial in enhancing their wellbeing and physical health [42, 43], consequently maintaining their cognitive functions [16] and reducing the odds of FoF [17–19]. Thus, it can be inferred that good social support is valuable for eliminating the FoF in the elderly, irrespective of whether they suffer from cognitive impairment or not.

### Strengths and limitations

To the best of our knowledge, the current study is the first study conducted in Vietnam to assess the association between FoF and cognitive function in the elderly according to their social support status. The study was conducted on a large representative sample of Vietnamese elderly, with a high response rate and an appropriate sampling method. FoF, perceived social support and cognitive function were measured using standardized and validated scales. However, being a cross-sectional study, no causal inference could be drawn and the results should therefore be interpreted with caution. A further longitudinal study is needed to investigate the two-way associations between the FoF and other factors. In addition, it is possible that the number of previous falls was underreported, because there was no direct question addressing this, and a history of fall was explored based on the types of bodily injury experienced by the participants during the last 12 months. However, we still found a significant association between a history of fall and a high FoF.

Given the fact that the coefficient of determination Pseudo R^2^ indicated that the independent variables explained 31 and 36% of variance of FoF in model 1 and model 2 respectively, results from this study should be interpreted with caution. However, the Goodness of Fit test suggested that these models were a good fit to the data as *p* = 0.6 (> 0.05, model 1) and *p* = 0.7 (> 0.05, model 2). Future studies are encouraged to consider other factors such as living conditions, psychology or physiological function in the research components.

### Implications

Our results highlight the important implications of the FoF for the vulnerable elderly living in the community. Although cognitive or functional impairment in older adults is inevitable with advancing age, factors such as the social support status are modifiable. Despite the fact that from a cultural perspective, Vietnamese elderly are respected and well supported, more than a half of the elderly (423/725) in this study perceived low or moderate level of social support in this study. With the recognition of this emerging issue, identification of isolated elderly in the community might be one of effective approaches for addressing individuals with a high risk of FoF. This could be done in practice by keeping close communication with local organizations such as Associations of the Elderly at community level, community health centers or provincial People’s Committees. Therefore, involvement of caregivers and stakeholders are important in improving the social support status of the elderly, by developing public programs focusing on social relationships and interactions, along with strengthening of the infrastructure in community to encourage more participation from the elderly.

## Conclusions

In summary, our study confirmed a high prevalence of a high FoF in an aging Vietnamese, and a number of factors associated with a FoF were identified. Our findings also indicated that cognitive impairment exerted differential effects on the likelihood of a high FoF in the elderly depending on the perceived social support levels. These findings are expected to be useful not only to screen for people who may benefit from fall-related programs, but for health care providers to devise well-targeted strategies towards prevention of falls and provide satisfactory interventions for the elderly residing in the community.

## Supplementary information

**Additional file 1.** Fully adjusted model to determine the associations between the participants’ characteristics and the fear of falling.

**Additional file 2.** Mean MMSE score of participants of low-moderate social support level and high social support level.

## Data Availability

Datasets used for this study are not publicly shared due to participants’ confidentiality, but are accessible from Institute for Community Health Research, College of Medicine and Pharmacy, Hue University on reasonable request for research.

## Abbreviations

CI: Confidence interval
OR: Odds ratio
AOR: Adjusted odd ratio
FoF: Fear of falling
FES-I: Fall Efficacy Scale-International
MMSE: Mini-Mental State Examination
IADL: Instrumental Activities of Daily Living Scale
BADL: Barthel Activities of Daily Living index
MSPSS: Multidimensional Scale of Perceived Social Support
