# Supplementary material for: Fear of falling and cognitive impairment in elderly with different social support levels: findings from a community survey in Central Vietnam
Source: BMC Geriatr. 2020 Apr 16;20:141. doi: 10.1186/s12877-020-01533-8 (PMC7164140; doi:10.1186/s12877-020-01533-8)
Supplement: Supplementary file 1 — Additional file 1. Fully adjusted model to determine the associations between the participants’ characteristics and the fear of falling. [file 12877_2020_1533_MOESM1_ESM.pdf]

Additional table 1:

Fully adjusted model to determine the associations between participants' characteristics and the fear of falling

		Fear of falling
		AOR[1] (95% CI)
Gender	Male	1
	Female	1.88 (1.20-2.95)**
Age (yr)	60-69	1
	70-79	1.28 (0.82-1.99)
	≥ 80	1.60 (0.93-2.74)
Marital status	Married	1
	Single or formerly married	0.96 (0.61-1.51)
History of fall	No	1
	Yes	3.83 (1.75-8.38)**
Arthritis	No	1
	Yes	0.91 (0.60-1.37)
Hypertension	No	1
	Yes	1.33 (0.92-1.94)
Limitation of the IADL	No	1
	Yes	2.49 (1.55-4.00)***
Limitation of the BADL	No	1
	Yes	2.65 (1.44-4.88)**
Visual ability	No difficulty	1
	Difficulty	1.60 (1.09-2.35)*
Walking ability	No difficulty	1
	Difficulty	3.24 (2.04-5.16)***
Cognitive function	Normal	1
	Impairment [2]	2.07 (1.19 –3.59)*

[1]AOR: Adjusted odd ratio; 95% CI = 95% Confidence interval

Adjusted for age, gender, marital status, history of fall, arthritis, hypertension, limitation of the IADL, limitation of the BADL, visual ability, walking ability and cognitive function.

[2] Cognitive impairment was defined by a score lower than the cut-off score of 23.

*p < 0.05; **p < 0.01; ***p < 0.001
