# Supplementary material for: Fear of falling and cognitive impairment in elderly with different social support levels: findings from a community survey in Central Vietnam
Source: BMC Geriatr. 2020 Apr 16;20:141. doi: 10.1186/s12877-020-01533-8 (PMC7164140; doi:10.1186/s12877-020-01533-8)
Supplement: Supplementary file 2 — Additional file 2. Mean MMSE score of participants of low-moderate social support level and high social support level. [file 12877_2020_1533_MOESM2_ESM.pdf]

Additional table 2:

Mean MMSE score of participants of low-moderate social support and high social support level

	Low-moderate social support level (n = 423)	High social support level (n = 302)	p	95% CI	
				Lower	Upper
MMSE mean score	25.46	26.59	0.003	-1.90	-0.38
